# Supplementary material for: Identification of a RAB32-LRMDA-Commander membrane trafficking complex reveals the molecular mechanism of human oculocutaneous albinism type 7
Source: Nat Commun. 2025 Oct 2;16:8794. doi: 10.1038/s41467-025-63855-8 (PMC12491506; doi:10.1038/s41467-025-63855-8)

SUPPLEMENTARY INFORMATION

Identification of a RAB32-LRMDA-Commander membrane trafficking complex reveals the molecular mechanism of human oculocutaneous albinism type 7

Rebeka Butkovič[1,*], Michael D. Healy[2], Cecilia de Heus[3], Alexander P. Walker[1], Wyatt Beyers[4], Kerrie E. McNally[5], Philip A. Lewis[6], Kate J. Heesom[6], Nalan Liv[3], Judith Klumperman[3], Santiago Di Pietro[4], Brett M. Collins[2], Peter J. Cullen[1,*]

[1]School of Biochemistry, Faculty of Life Sciences, Biomedical Sciences Building, University of Bristol, Bristol BS8 1TD, UK.

[2]Centre for Cell Biology of Chronic Disease, Institute for Molecular Biosciences, The University of Queensland, St. Lucia, QLD 4072, Australia.

[3]Center for Molecular Medicine, University Medical Center Utrecht, Utrecht University, 3584 CX Utrecht, The Netherlands.

[4]Department of Biochemistry and Molecular Biology, Colorado State University, 111 MRC Building, 1870 Campus Delivery, Fort Collins, Colorado 80523-1870, USA.

[5]MRC Laboratory of Molecular Biology, Cambridge, UK.

[6]Bristol Proteomics Facility, School of Biochemistry, Faculty of Life Sciences, Biomedical Sciences Building, University of Bristol, Bristol BS8 1TD, UK.

[*]Co-corresponding authors: rebeka.butkovic@bristol.ac.uk; pete.cullen@bristol.ac.uk

This file contains 5 supplementary figures and figure legends.

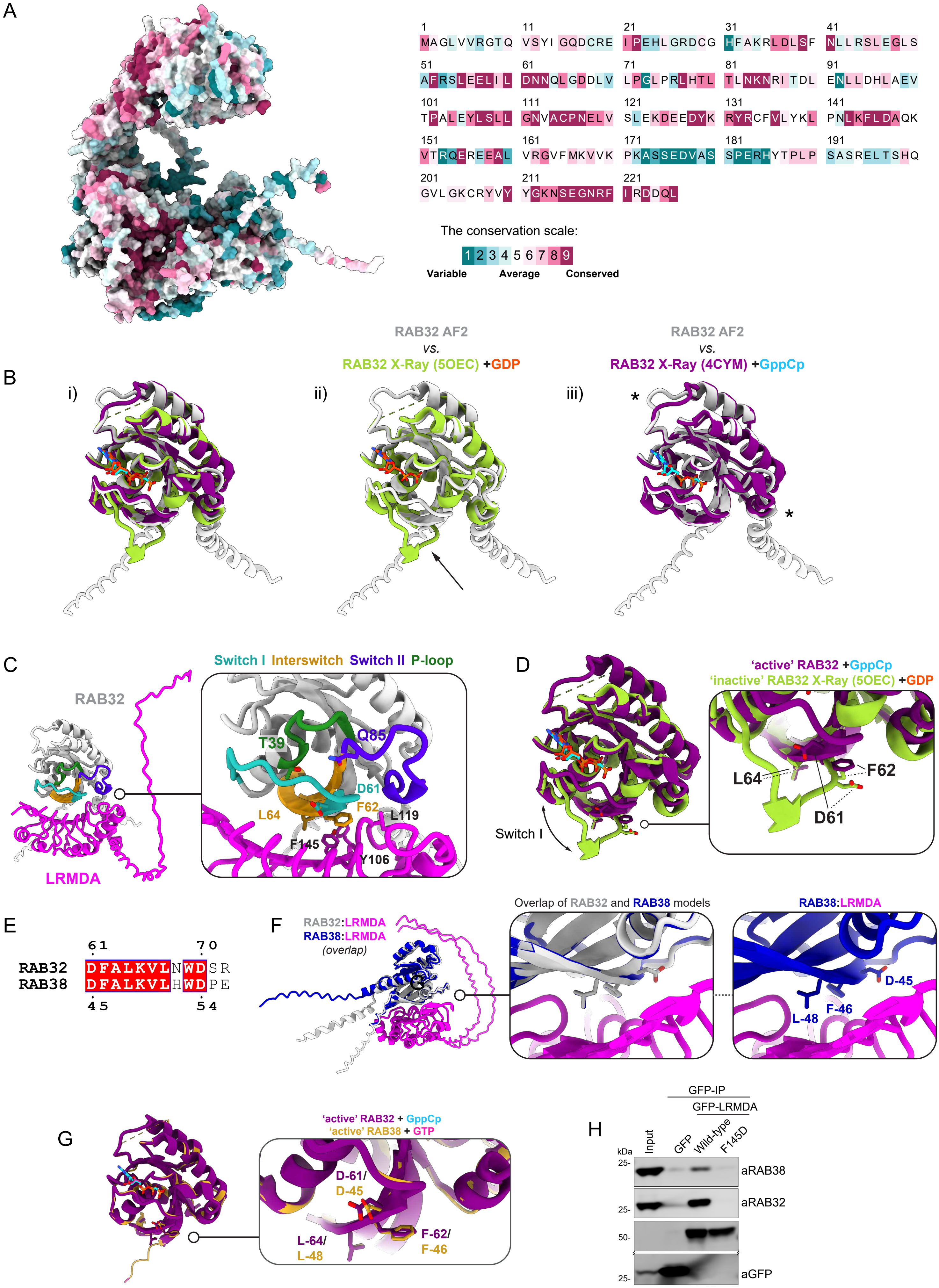

A
1 MAGLVVRGTQ
11 VSYIGQDCRE
21 IPEHLGRDCG
31 HFAKRLDLSF
41 NLLRSLEGLS
51 AFRSLEELIL
61 DNNQLGDDLV
71 LPGLPRLHTL
81 TLNKNRITDL
91 ENLLDHLAEV
101 TPALEYLSLL
111 GNVACPNELV
121 SLEKDEEDYK
131 RYRCFVLYKL
141 PNLKFLDAQK
151 VTRQEREEAL
161 VRGVFMKVVK
171 PKASSEDVAS
181 SPERHYTPLP
191 SASRELTSHQ
201 GVLGKCRYVY
211 YGKNSEGNRF
221 IRDDQL
The conservation scale:
1 2 3 4 5 6 7 8 9
Variable
Average
Conserved
B
i)
ii)
iii)
RAB32 AF2
vs.
RAB32 X-Ray (5OEC) +GDP
RAB32 AF2
vs.
RAB32 X-Ray (4CYM) +GppCp
*
*
C
Switch I Interswitch Switch II P-loop
RAB32
LRMDA
T39
Q85
D61
F62
L64
L119
F145
Y106
D
'active' RAB32 +GppCp
'inactive' RAB32 X-Ray (5OEC) +GDP
Switch I
L64
F62
D61
E
61
70
RAB32 DFALKVLNWDSR
RAB38 DFALKVLHWDPE
45
54
F
RAB32:LRMDA
RAB38:LRMDA
(overlap)
Overlap of RAB32 and RAB38 models
RAB38:LRMDA
D-45
F-46
L-48
G
'active' RAB32 + GppCp
'active' RAB38 + GTP
D-61/
D-45
L-64/
L-48
F-62/
F-46
H
GFP-IP
GFP-LRMDA
Input
GFP
Wild-type
F145D
kDa
25-
aRAB38
25-
aRAB32
50-
25-
aGFP

Supplementary Figure 1:

A) Evolutionary conservation of RAB32-LRMDA-Retriever assembly (Left) and LRMDA (Right) as determined by ConSurf tool. The orientation of model matches the orientation of the model shown in Figure 2A.

B) Comparsion of AlphaFold2-predicted RAB32 conformation with experimentally resolved structures of (ii) inactive GDP-bound (PDB: 5OEC) [21], or (iii) active GppCp-bound RAB32 (GppCp is a non-hydrolisable GTP analog; PDB: 4CYM) [23]. The depiction shows beter alignment with the active RAB32, and minor conformational differences are marked with stars. The conformation difference is more apparent in comparison to GDP-bound RAB32, where the change in position of Switch I region is highlighted with an arrow.

C) AlphaFold2-predicted RAB32:LRMDA assembly with annotation for the Switch I, Interswitch, Switch II and P-loop regions of RAB32 at the interface with LRMDA, and the location of residues, relevant for the interaction.

D) Comparison of active and inactive-RAB32 from panel (B) shows the localised change in flexibility of Switch I that occurs upon GTP binding. In inactive RAB32, residues that are important for LRMDA interaction (D61 and F62) are displaced.

E) Alignment of RAB32 and RAB38 showing the conservation of residues D-61, F-62 and L-64 (numbering for RAB32).

F) Alignment of AlphaFold-2 predicted models for the assembly of RAB32-LRMDA and RAB38-LRMDA.

G) Alignment of active RAB32 with active, GTP-bound, RAB38 (PDB: 6HDU) [24] shows the similarity in conformation of predicted interfacial residues.

H) MNT1 cells were lentivirally transduced with GFP, wild-type GFP-LRMDA or mutant GFP-LRMDA F145D, and used in GFP-immunoprecipitation experiment to probe the interaction with RAB32 and the related protein RAB38.

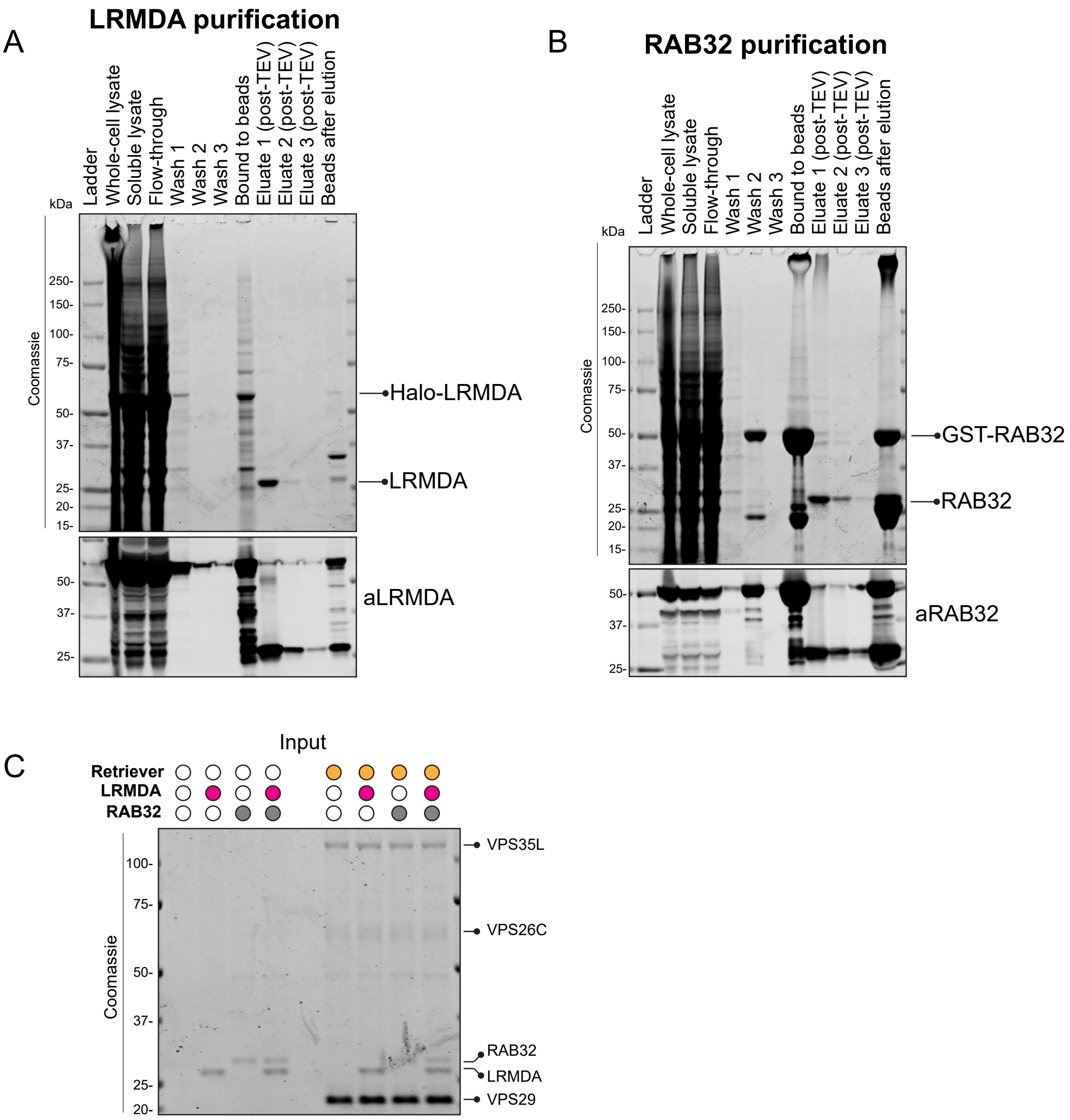
A
LRMDA purification
Ladder
Whole-cell lysate
Soluble lysate
Flow-through
Wash 1
Wash 2
Wash 3
Bound to beads
Eluate 1 (post-TEV)
Eluate 2 (post-TEV)
Eluate 3 (post-TEV)
Beads after elution
kDa
250-
150-
100-
75-
50-
37-
25-
20-
15-
Coomassie
Halo-LRMDA
LRMDA
aLRMDA
B
RAB32 purification
GST-RAB32
RAB32
aRAB32
C
Input
Retriever
LRMDA
RAB32
VPS35L
VPS26C
RAB32
LRMDA
VPS29

Supplementary Figure 2:

A) Halo-LRMDA was expressed in insect cells, and purified using HaloLink resin. TEV protease was used to remove the Halo Tag.

B) GST-RAB32 was expressed in insect cells, and purified using Glutathione Sepharose beads. TEV protease was used to remove the GST Tag.

C) Inputs for the experiment shown in Figure 3E.

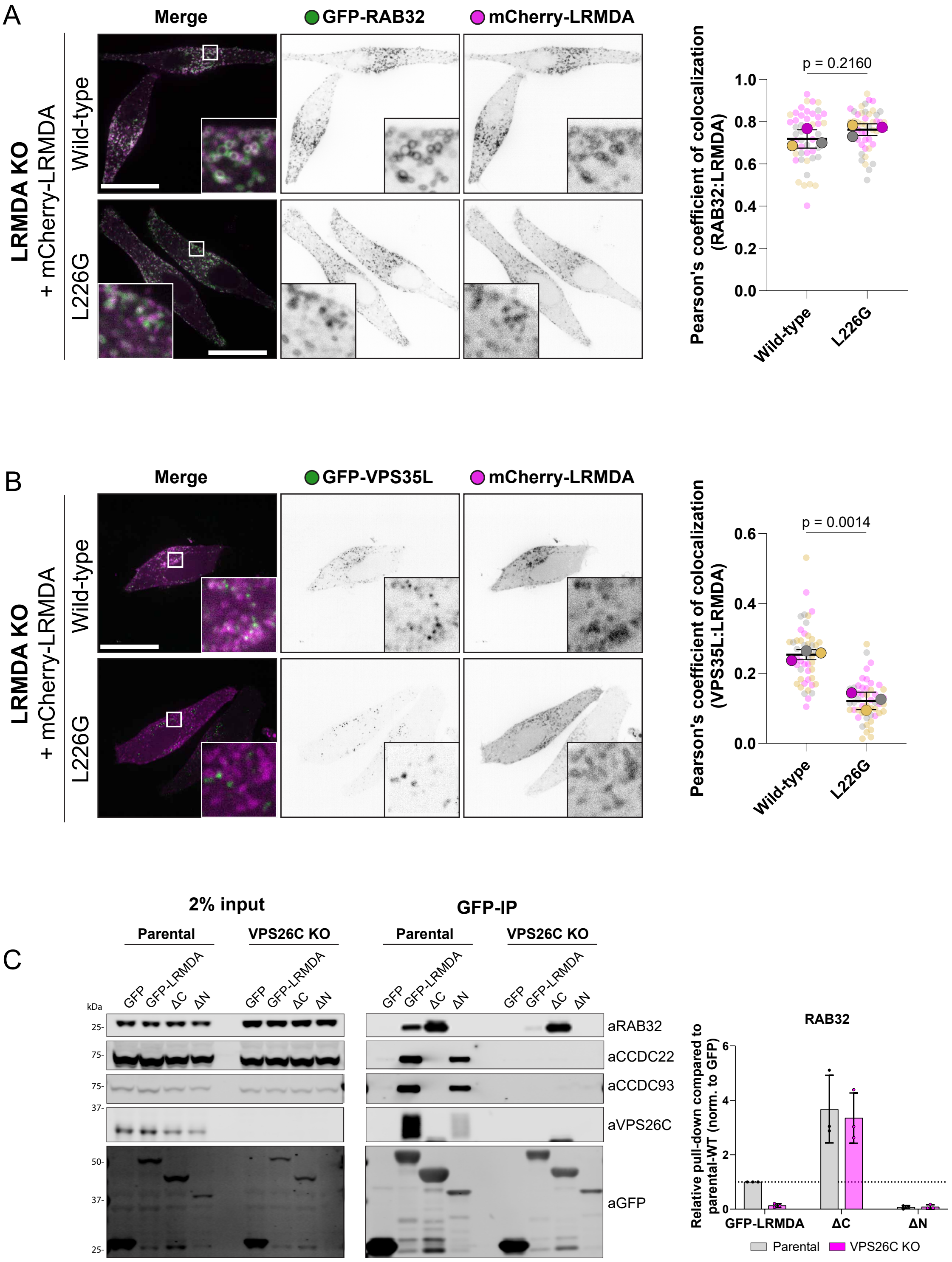
A
Merge
GFP-RAB32
mCherry-LRMDA
LRMDA KO
+ mCherry-LRMDA
Wild-type
L226G
p = 0.2160
Pearson's coefficient of colocalization (RAB32:LRMDA)
1.0
0.8
0.6
0.4
0.2
0.0
Wild-type
L226G
B
Merge
GFP-VPS35L
mCherry-LRMDA
LRMDA KO
+ mCherry-LRMDA
Wild-type
L226G
p = 0.0014
Pearson's coefficient of colocalization (VPS35L:LRMDA)
0.6
0.4
0.2
0.0
Wild-type
L226G
C
2% input
GFP-IP
Parental
VPS26C KO
Parental
VPS26C KO
GFP
GFP-LRMDA
ΔC
ΔN
kDa
25-
75-
75-
37-
50-
37-
25-
aRAB32
aCCDC22
aCCDC93
aVPS26C
aGFP
RAB32
Relative pull-down compared to parental-WT (norm. to GFP)
6
4
2
0
GFP-LRMDA
ΔC
ΔN
Parental
VPS26C KO

Supplementary Figure 3:

A) MNT1 LRMDA KO cells were co-transfected with GFP-RAB32 and wild-type mCherry-LRMDA or mutant mCherry-LRMDA L226G. Live cells were observed using spinning disk microscope to assess the spatial organisation of LRMDA and RAB32. Scale bar corresponds to 20 µm. n = 3 (wild-type GFP-LRMDA – 47 cells; GFP-LRMDA L226G – 51 cells), large circles represent averages of each independent experiment, and small circles the data for individual cells, colouring corresponds to different independent replicates. Two-tailed unpaired t-test on data for replicate averages, data presented as mean values and error bars represent s.d.

B) MNT1 LRMDA KO cells were co-transfected with GFP-VPS35L and wild-type mCherry-LRMDA or mutant mCherry-LRMDA L226G. Live cells were observed using spinning disk microscope to assess the spatial organisation of LRMDA and VPS35L. Scale bar corresponds to 20 µm. n = 3 (wild-type GFP-LRMDA – 47 cells; GFP-LRMDA L226G – 46 cells), large circles represent averages of each independent experiment, and small circles the data for individual cells, colouring corresponds to different independent replicates. Two-tailed unpaired t-test on data for replicate averages, data presented as mean values and error bars represent s.d.

C) Parental or VPS26C KO HeLa cells were transfected with GFP-LRMDA or truncated forms of GFP-LRMDA, and the lysates were used in GFP-trap experiments to analyze the association with Retriever complex or RAB32. n = 3, 2-way ANOVA with Šídák's multiple comparisons test, data presented as mean values and error bars represent s.d.; n.s. denotes changes with $p > 0.05$.

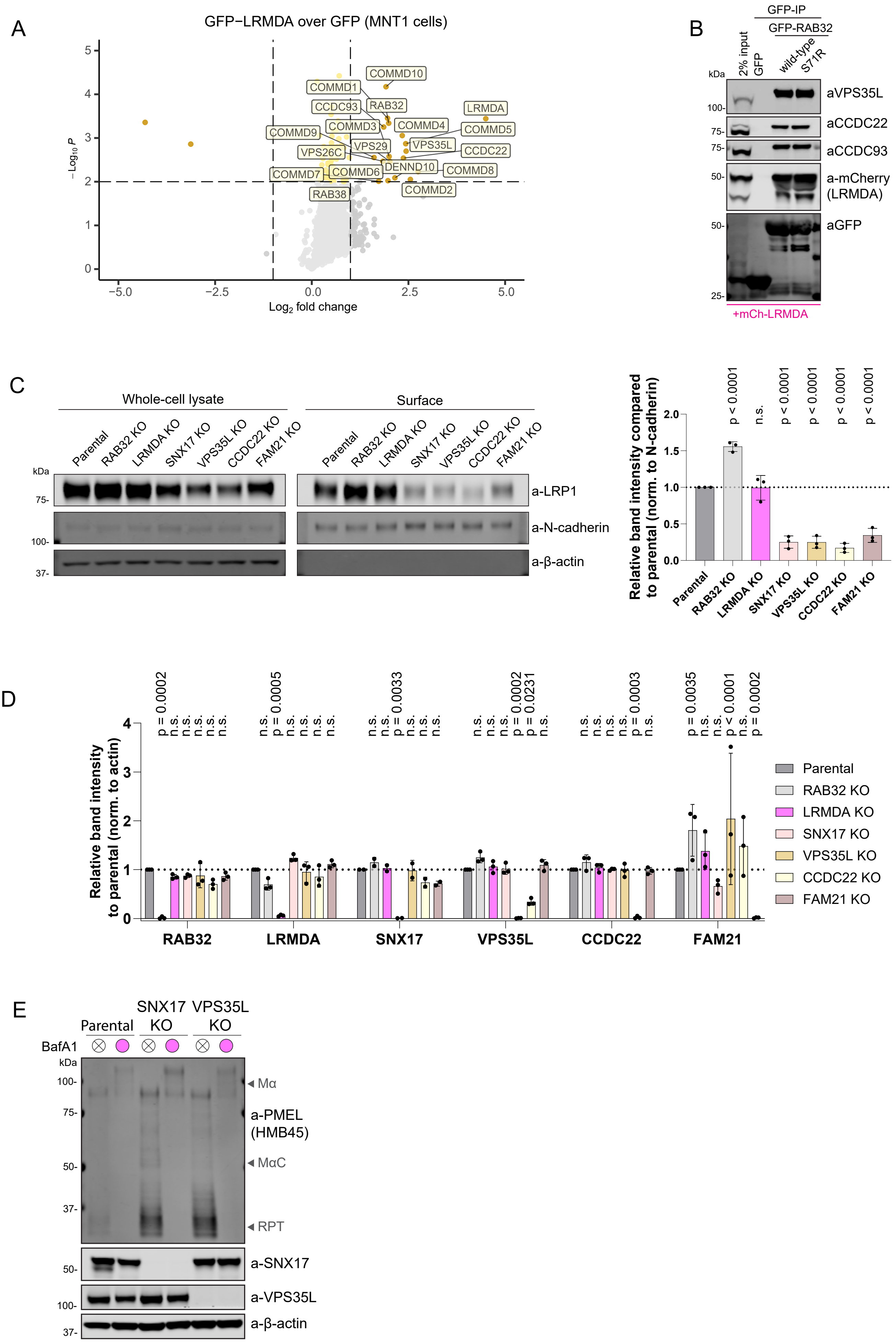
A
GFP−LRMDA over GFP (MNT1 cells)
COMMD10
COMMD1
CCDC93
RAB32
LRMDA
COMMD9
COMMD3
COMMD4
COMMD5
VPS29
VPS35L
VPS26C
CCDC22
DENND10
COMMD7
COMMD6
COMMD8
RAB38
COMMD2
$-Log_{10} P$
Log_2 fold change
B
GFP-IP
GFP-RAB32
2% input
GFP
wild-type
S71R
kDa
aVPS35L
aCCDC22
aCCDC93
a-mCherry
(LRMDA)
aGFP
+mCh-LRMDA
C
Whole-cell lysate
Surface
Parental
RAB32 KO
LRMDA KO
SNX17 KO
VPS35L KO
CCDC22 KO
FAM21 KO
a-LRP1
a-N-cadherin
a-β-actin
Relative band intensity compared to parental (norm. to N-cadherin)
p < 0.0001
n.s.
p < 0.0001
p < 0.0001
p < 0.0001
p < 0.0001
D
Relative band intensity to parental (norm. to actin)
p = 0.0002
p = 0.0005
p = 0.0033
p = 0.0002
p = 0.0231
p = 0.0003
p = 0.0035
p < 0.0001
p = 0.0002
n.s.
RAB32
LRMDA
SNX17
VPS35L
CCDC22
FAM21
Parental
RAB32 KO
LRMDA KO
SNX17 KO
VPS35L KO
CCDC22 KO
FAM21 KO
E
Parental
SNX17 KO
VPS35L KO
BafA1
kDa
Mα
a-PMEL
(HMB45)
MαC
RPT
a-SNX17
a-VPS35L
a-β-actin

Supplementary Figure 4:

A) Volcano plot showing the enrichment of proteins in GFP-LRMDA pulldown in MNT1 cells, compared to the GFP sample. N=3, paired t-test. All significantly enriched proteins with $p < 0.01$ and Log2 fold change > 1 are labelled, along with RAB38 due to its biological relevance. Proteins with FDR < 0.05 are depicted with triangles.

B) HEK293T cells were co-transfected with mCherry-LRMDA and GFP, GFP-RAB32 or Parkinson's disease-causative mutant GFP-RAB32 S71R. The lysates were used in GFP-trap experiments to analyze the association with Retriever complex or LRMDA.

C) Cell surface proteins were biotinylated in parental, RAB32 KO, LRMDA KO, SNX17 KO, VPS35L KO, CCDC22 KO and FAM21 KO MNT1 cells and enriched with streptavidin pull-down to analyze surface protein levels. The SNX17 KO, VPS35L KO, CCDC22 KO and FAM21 KO MNT1 cells display decreased surface levels of LRP1. The quantitative analysis of LRP1 band intensities is shown. n = 3, 1-way ANOVA with Dunnett's multiple comparison test, error bars represent s.d.

D) Protein levels in whole-cell lysates (RIPA lysis buffer) for the experiment shown in Figure 7B. n = 3, 2-way ANOVA with Dunnett's multiple comparison test, data presented as mean values and error bars represent s.d.; n.s. denotes changes with $p > 0.05$.

E) Protein levels in whole-cell lysates (RIPA lysis buffer) from parental or SNX17 KO or VPS35L KO cells treated with 100 nM BafA1 for 24h.

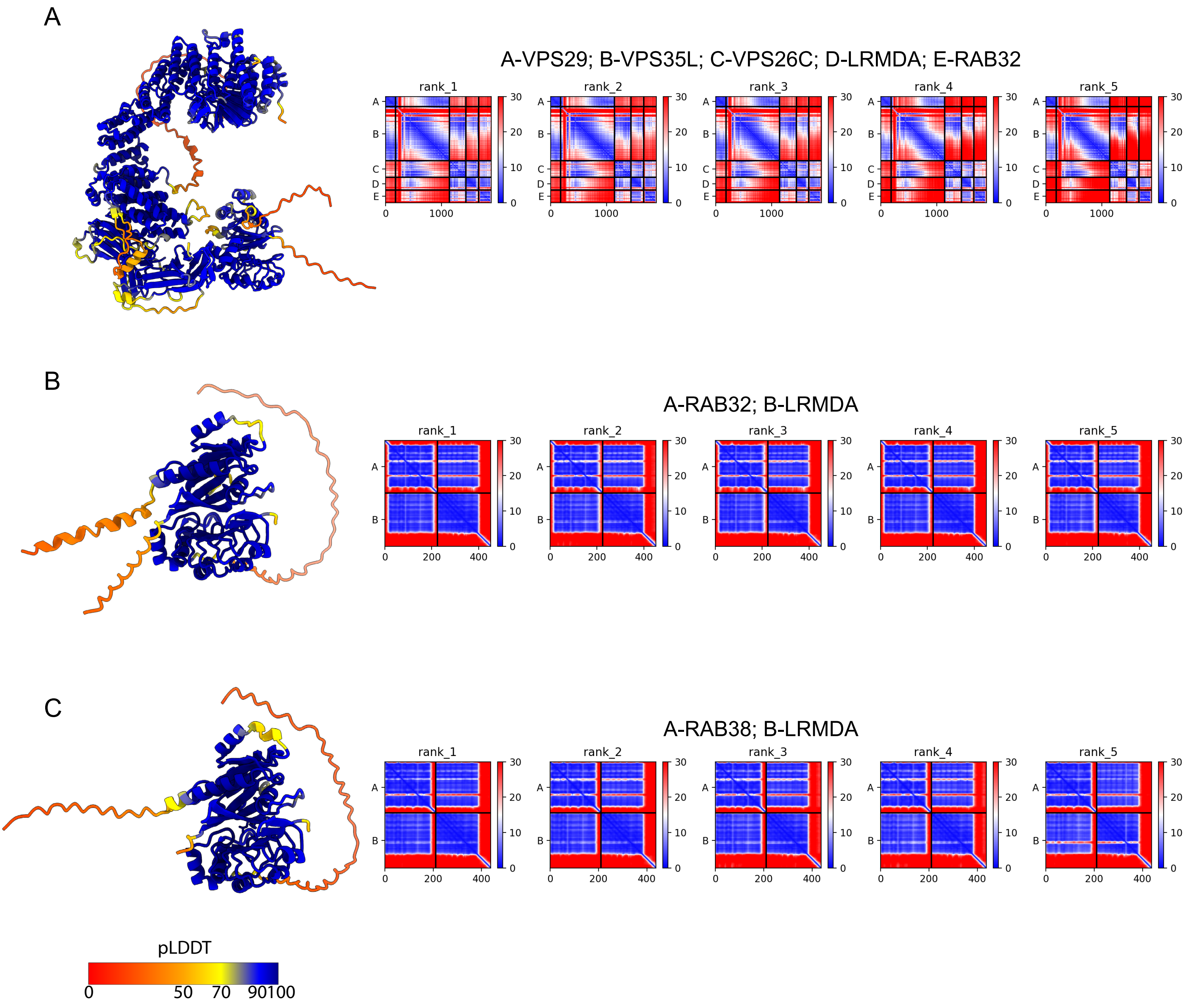

A
A-VPS29; B-VPS35L; C-VPS26C; D-LRMDA; E-RAB32
rank_1
rank_2
rank_3
rank_4
rank_5
B
A-RAB32; B-LRMDA
C
A-RAB38; B-LRMDA
pLDDT
0
50
70
90100

Supplementary Figure 5:

pLDDT scores and PAE plots for AlphaFold-2 predicted assemblies between LRMDA-RAB32-Retriever (A), LRMDA-RAB32 (B) and LRMDA-RAB38 (C).

UNCROPPED GELS AND BLOT SCANS FOR SUPPLEMENTARY FIGURES

Raw blots for Supplementary Figure 1

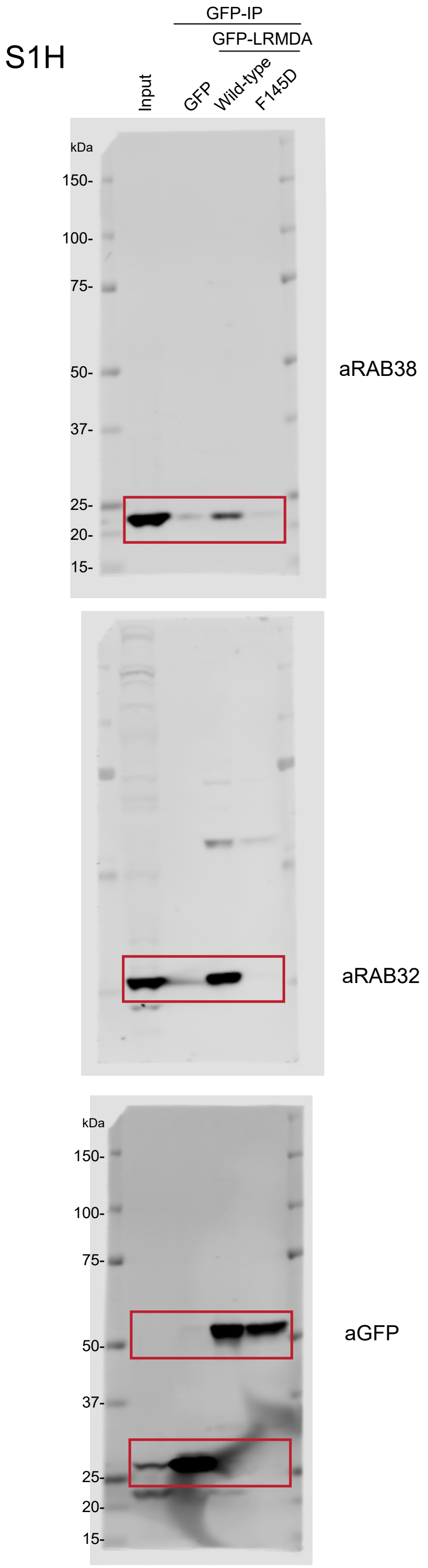

Raw blots for Supplementary Figure 2

S2A

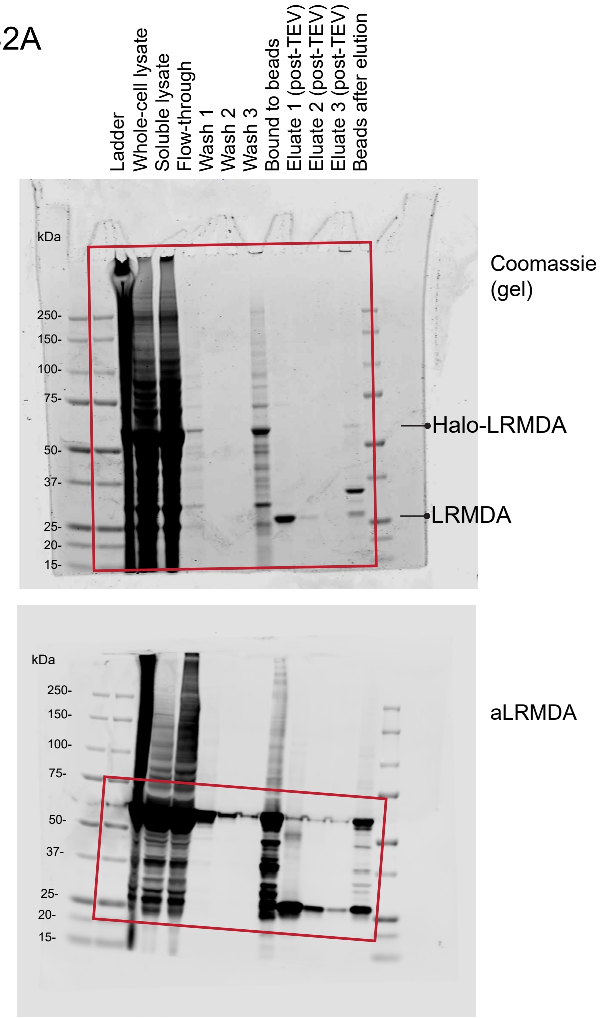

S2B

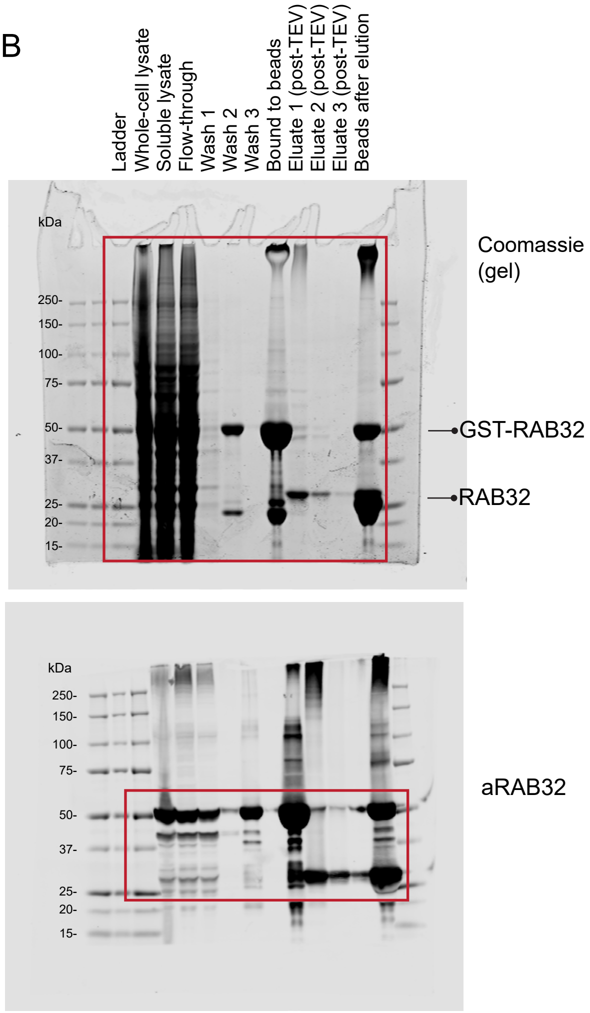

S2C

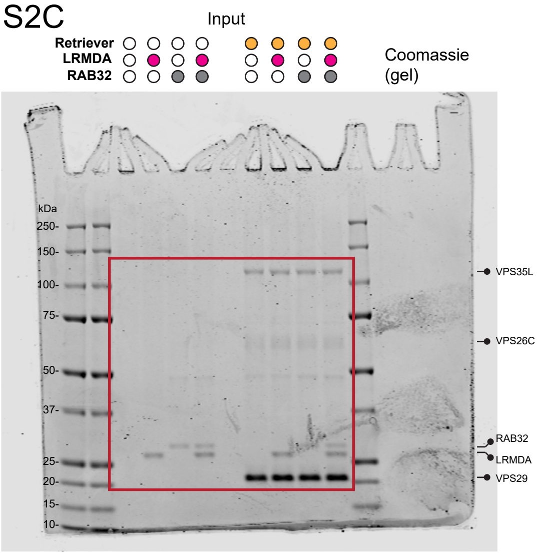

Raw blots for Supplementary Figure 3

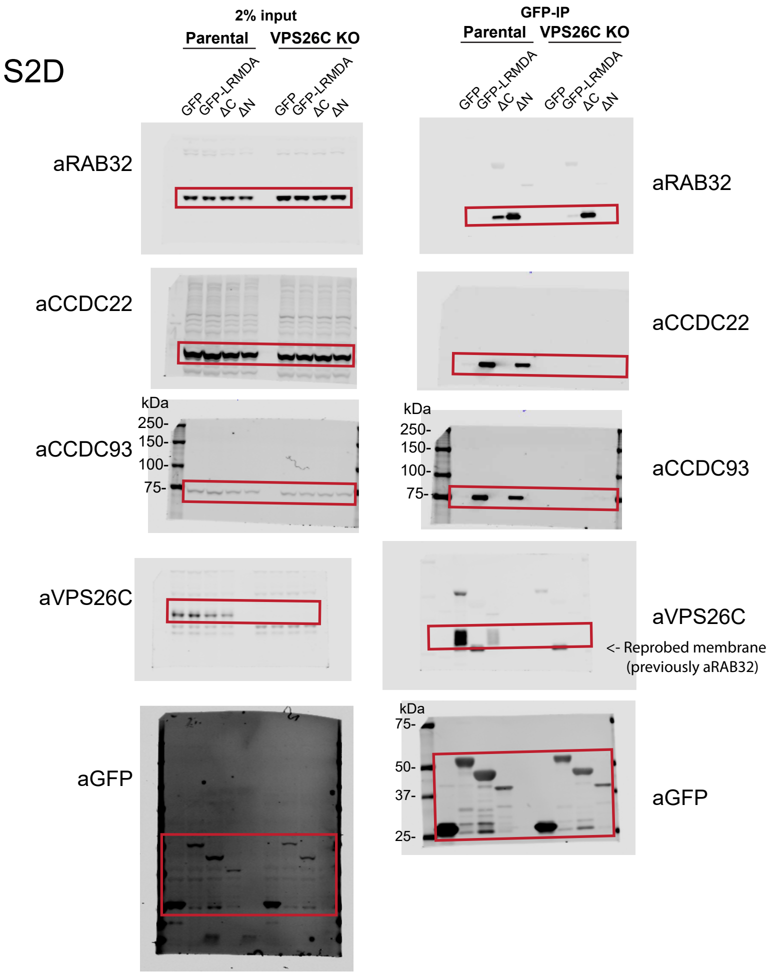

Raw blots for Supplementary Figure 4

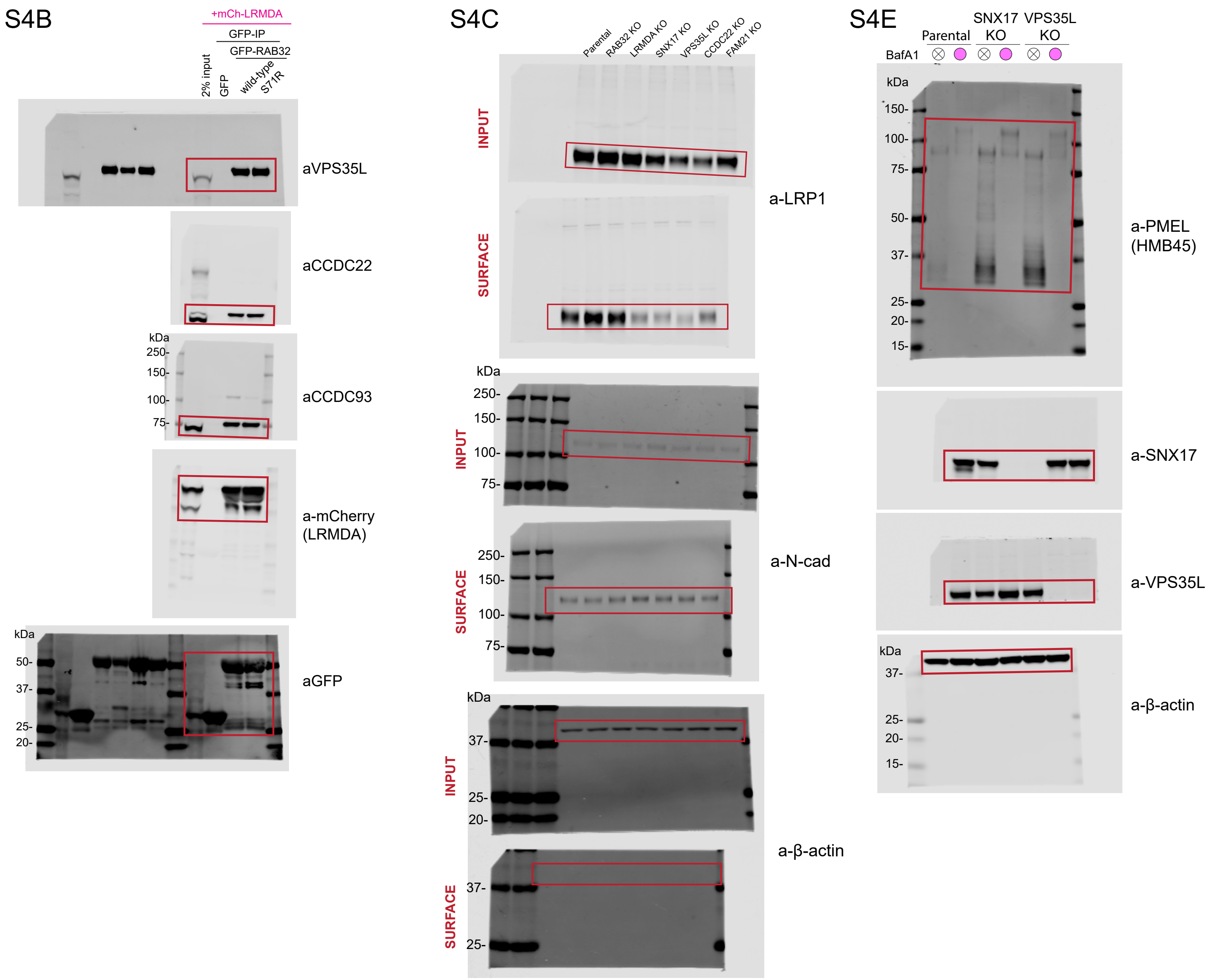

Supplement: Supplementary file 1 — Supplementary information [file 41467_2025_63855_MOESM1_ESM.pdf]
