# Supplementary material for: Identification of a RAB32-LRMDA-Commander membrane trafficking complex reveals the molecular mechanism of human oculocutaneous albinism type 7
Source: Nat Commun. 2025 Oct 2;16:8794. doi: 10.1038/s41467-025-63855-8 (PMC12491506; doi:10.1038/s41467-025-63855-8)
Supplement: Supplementary file 2 — Description of Additional Supplementary File [file 41467_2025_63855_MOESM2_ESM.pdf]

Description of Additional Supplementary File

Supplementary data 1 : Interactome analysis (proteomics)

Supplementary data 2 : cloning primer and site-directed mutagenesis primer sequences
