# Supplementary material for: Identification of a RAB32-LRMDA-Commander membrane trafficking complex reveals the molecular mechanism of human oculocutaneous albinism type 7
Source: Nat Commun. 2025 Oct 2;16:8794. doi: 10.1038/s41467-025-63855-8 (PMC12491506; doi:10.1038/s41467-025-63855-8)

Raw blots for Figure 1

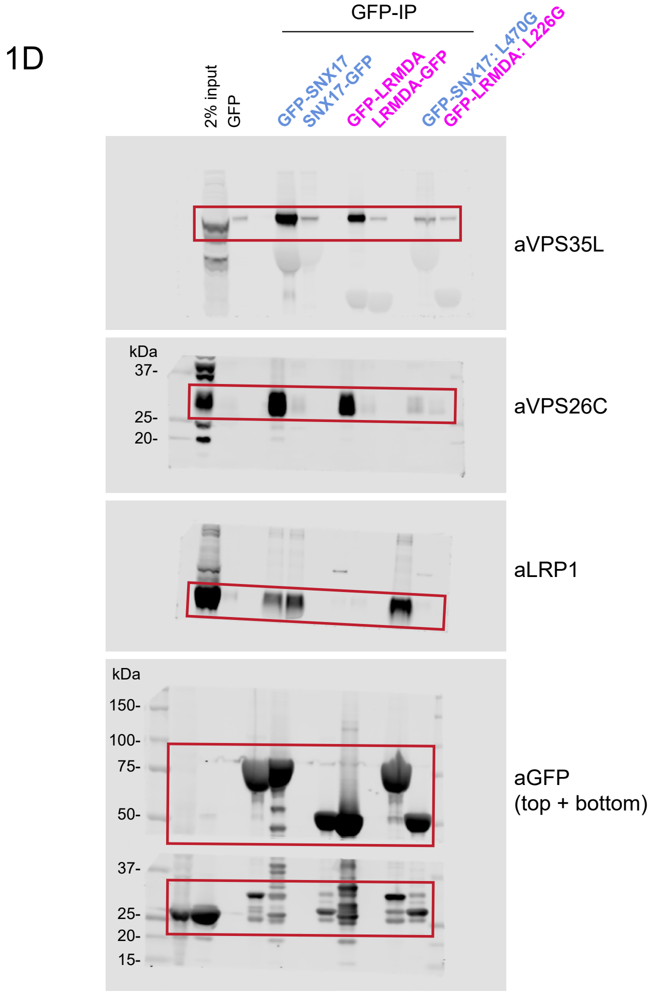

Raw blots for Figure 2

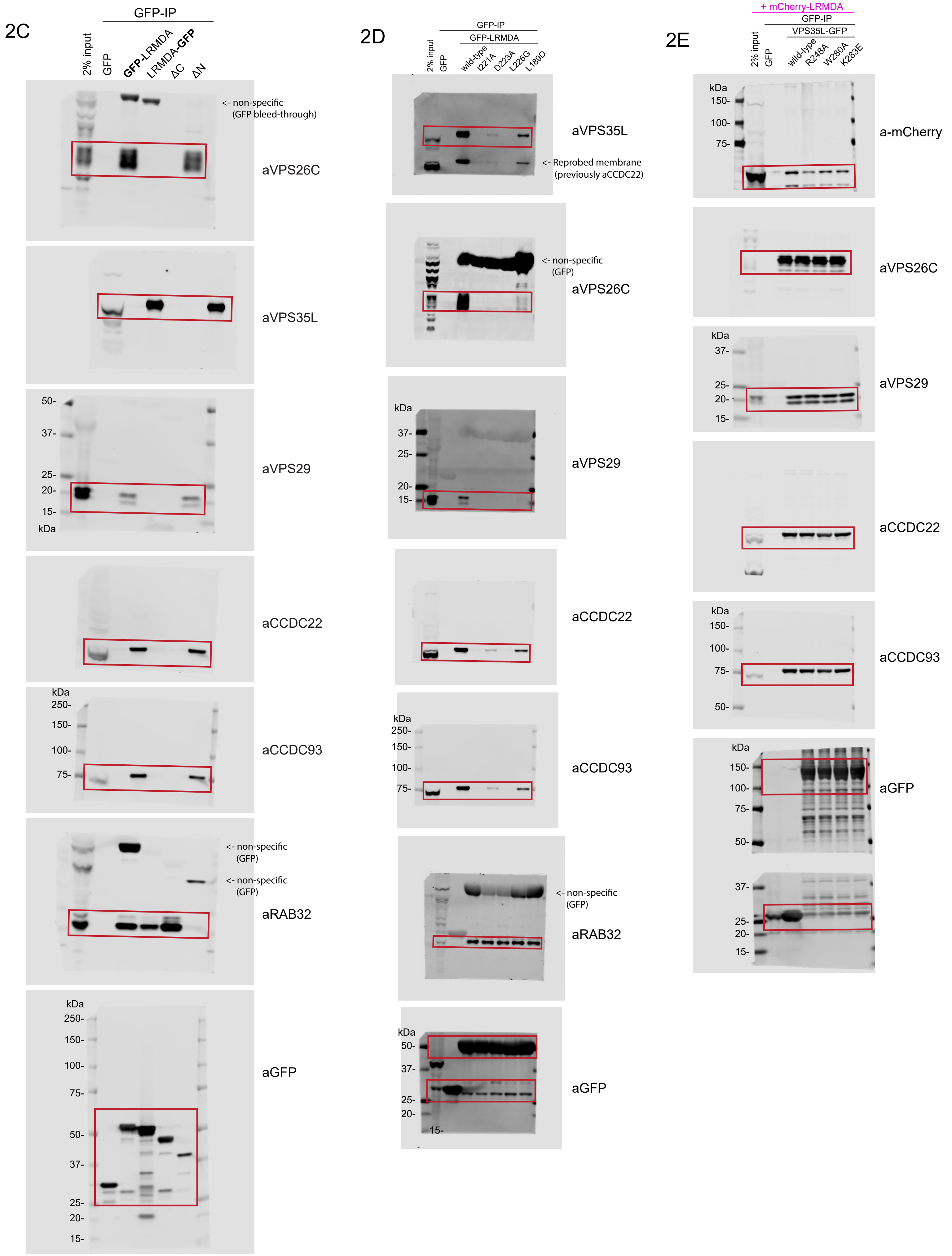

Raw blots for Figure 3

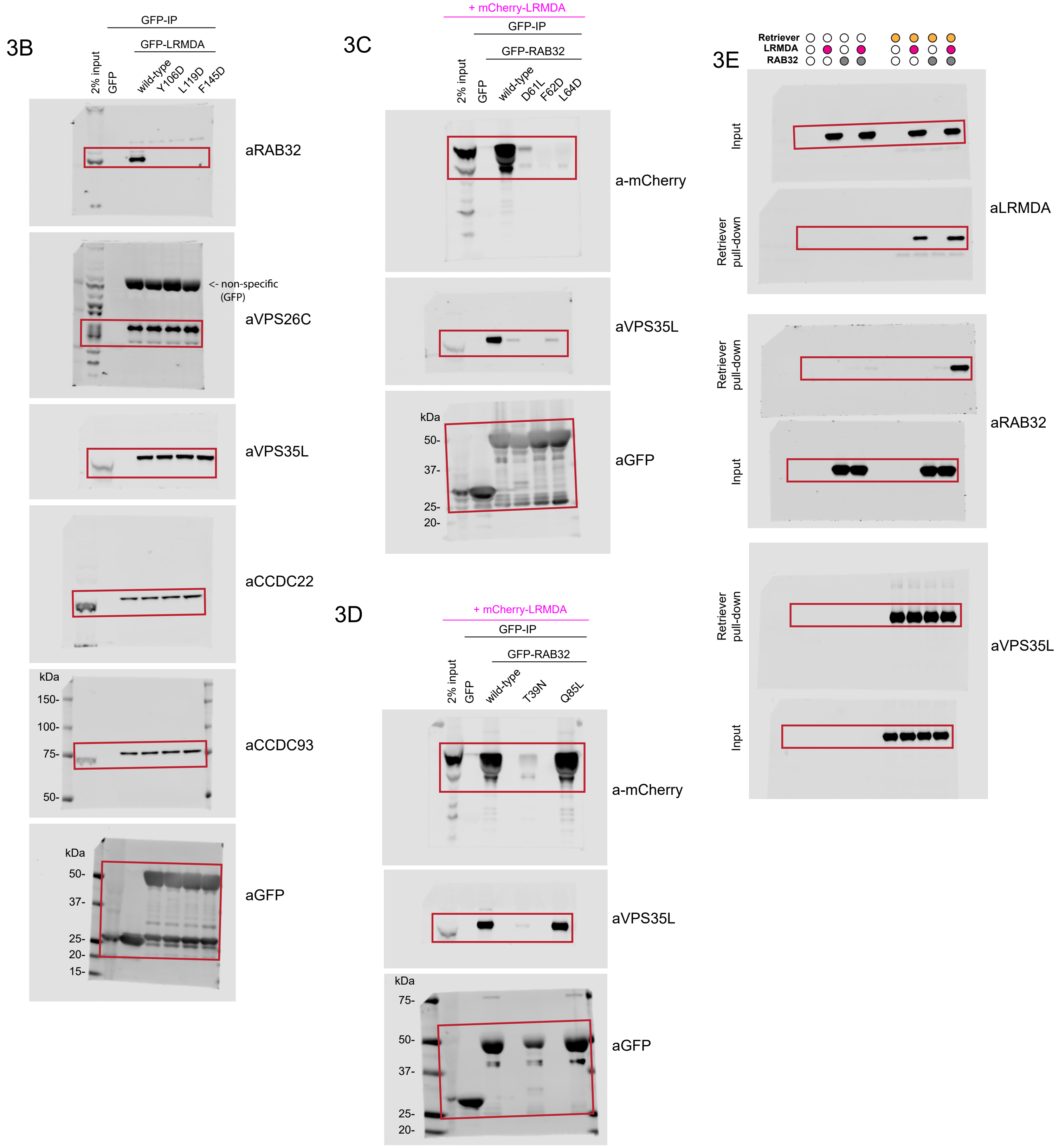

Raw blots for Figure 4

4E

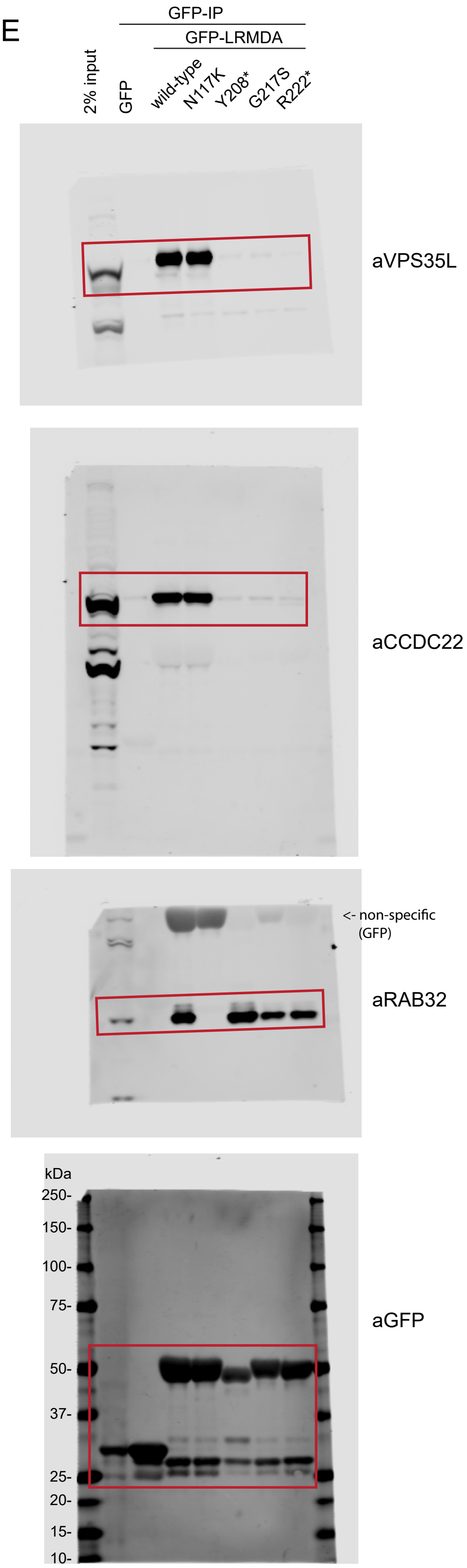

4F

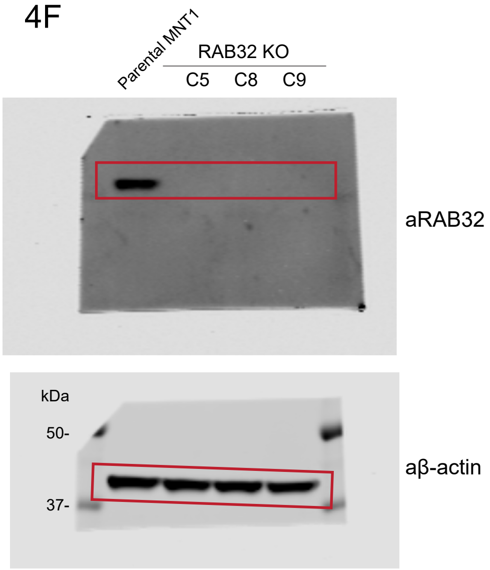

4I

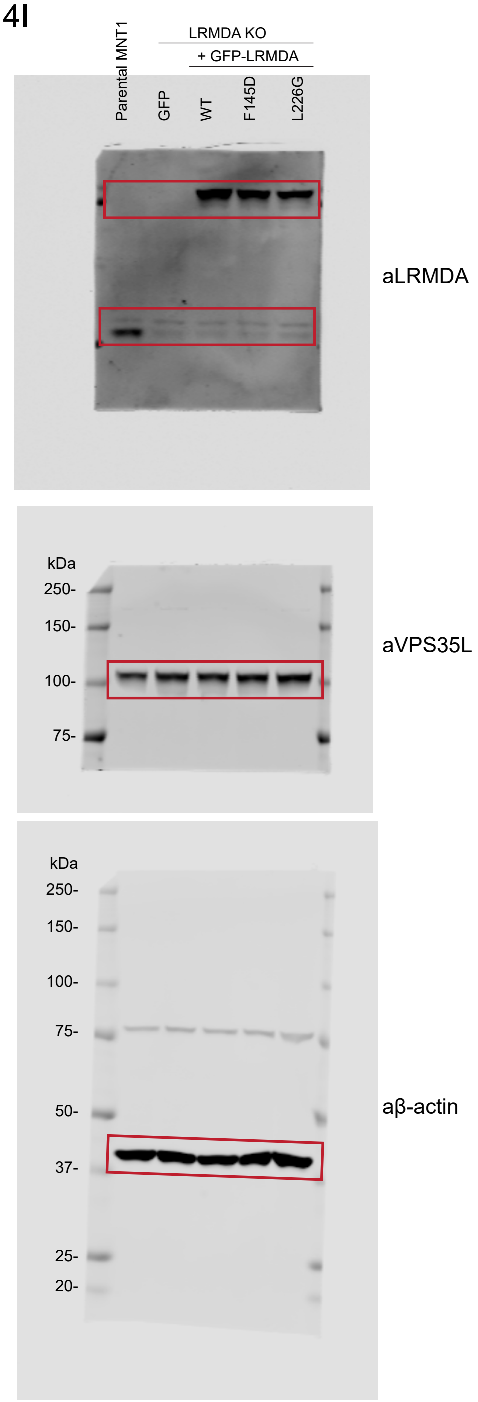

Raw blots for Figure 5

5A

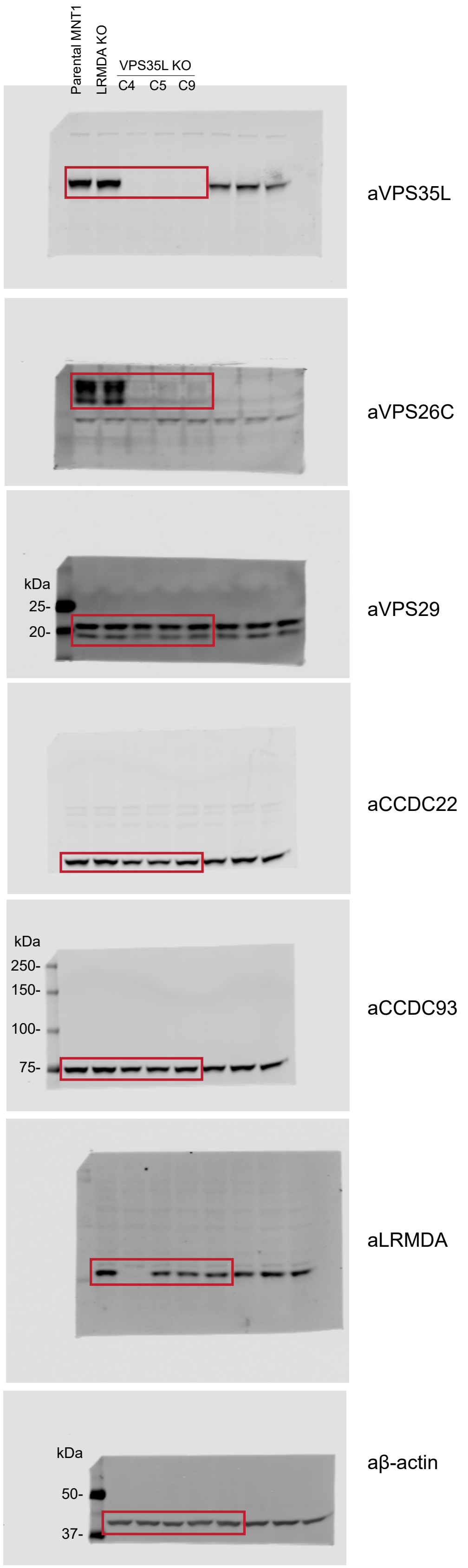

Raw blots for Figure 6

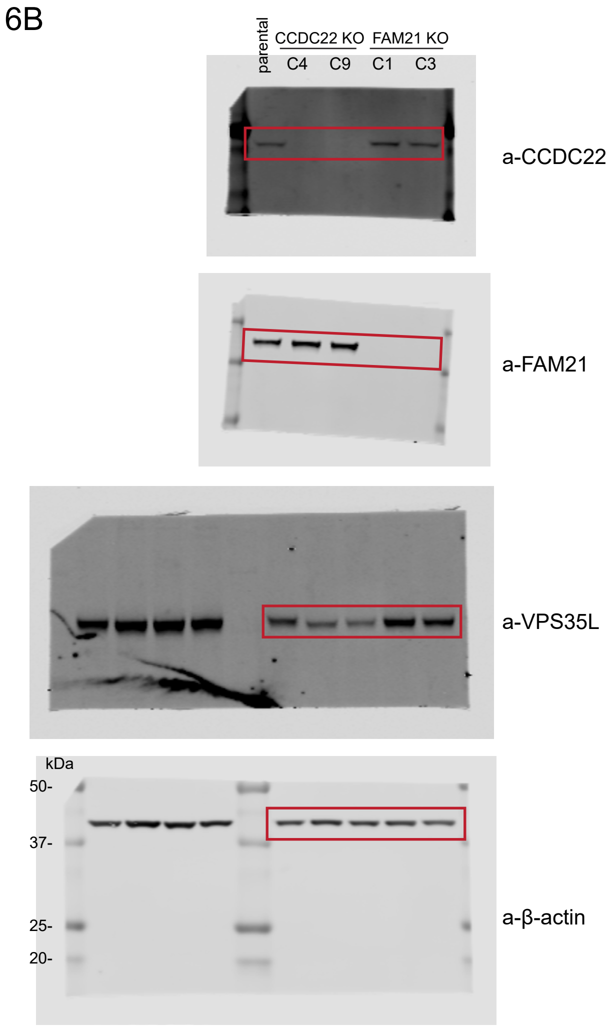

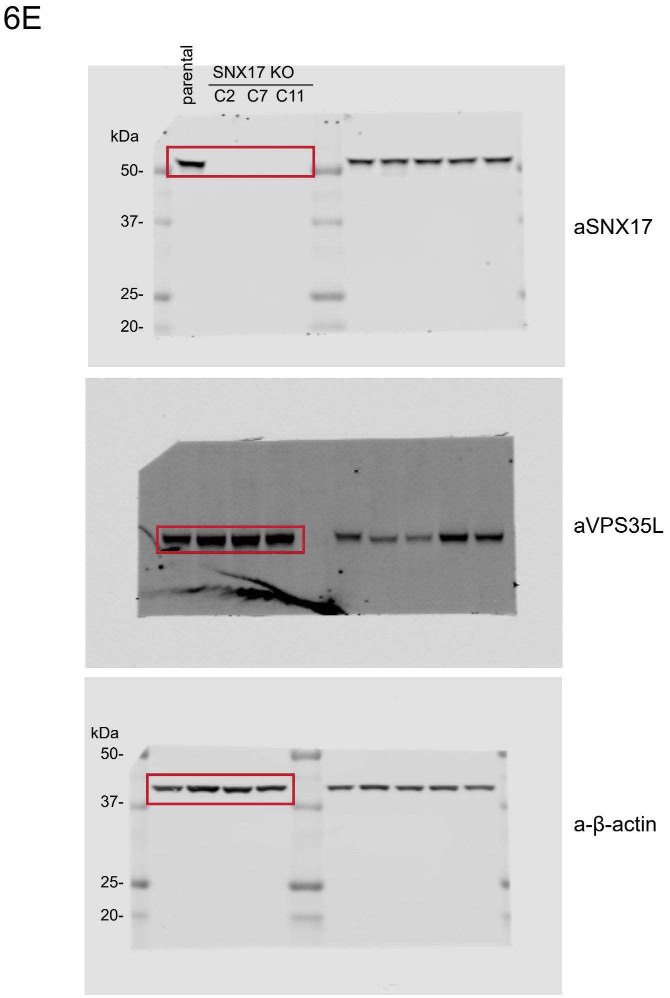

Raw blots for Figure 7

7B

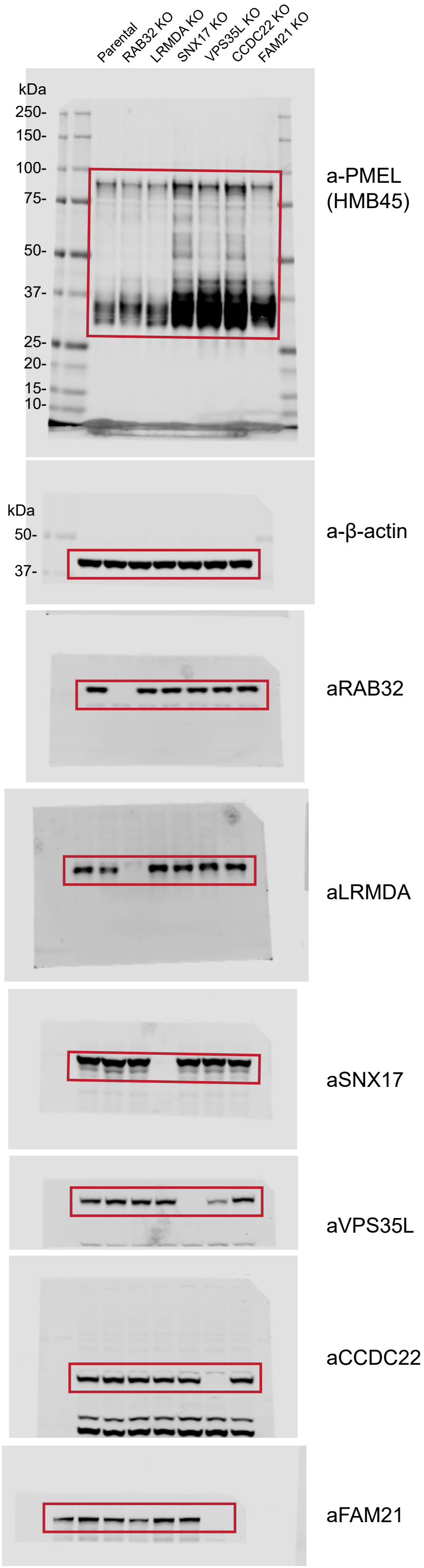

Supplement: Supplementary file 7 — Source Data [file 41467_2025_63855_MOESM7_ESM.zip › Source data.pdf]
